# Comparison of cardiothoracic surgery training in usa and germany

**DOI:** 10.1186/1749-8090-5-118

**Published:** 2010-11-26

**Authors:** Vakhtang Tchantchaleishvili, Suyog A Mokashi, Taufiek K Rajab, R Morton III Bolman, Frederick Y Chen, Jan D Schmitto

**Affiliations:** 1 Division of Cardiac Surgery, Brigham and Women's Hospital, Harvard Medical School, Boston, MA, USA; 2 Division of Cardiac, Thoracic and Vascular Surgery, University Hospital of Goettingen, Goettingen, Germany

## Abstract

**Background:**

Training of cardiothoracic surgeons in Europe and the United States has expanded to incorporate new operative techniques and requirements. The purpose of this study was to compare the current structure of training programs in the United States and Germany.

**Methods:**

We thoroughly reviewed the existing literature with particular focus on the curriculum, salary, board certification and quality of life for cardiothoracic trainees.

**Results:**

The United States of America and the Federal Republic of Germany each have different cardiothoracic surgery training programs with specific strengths and weaknesses which are compared and presented in this publication.

**Conclusions:**

The future of cardiothoracic surgery training will become affected by technological, demographic, economic and supply factors. Given current trends in training programs, creating an efficient training system would allow trainees to compete and grow in this constantly changing environment.

## Introduction

Cardiothoracic surgeons must possess a wide variety of technical and professional competencies. With time, cardiac operations are becoming increasingly difficult given aging patient population with more co-morbidities and increasingly severe coronary artery disease. On the other hand, training in cardiothoracic surgery is increasingly being restricted by work hour limitations. There are recent trends to reshape cardiothoracic surgery training to make it more efficient and productive. In this regard, it is very intersting and useful to examine various training systems globally. We decided to compare cardiothoracic surgery training system in the United States with the training system in Germany. Germany has one of the best developed cardiothoracic surgery training systems in the world and at the same time differs enough from U.S. training system to be considered for such a comparison.

## Methods

Available literature regarding cardiothoracic surgery training in the United States and Germany was reviewed by cardiothoracic surgeons in training and trained cardiothoracic surgeons from U.S. and Germany. Up-to-date publications by American Board of Thoracic Surgery (ABTS) and Accreditation Council for Graduate Medical Education (ACGME) were reviewed. Information about cardiothoracic surgery training in U.S.A. and Germany were divided in different aspects and qualitatively compared. Number of required cases and financial compensation in two countries were compared quantitatively. The term "cardiothoracic surgery" used in this manuscript refers to both cardiac and general thoracic surgery.

## Results

### Work hours restriction

Accredited residency programs in United States are restricted by 80 hours/week. German resident work-hours are restricted to 42 hours/week with additional hours on call, averaging 4-8 on call nights per month.

### Structure of Training

At this time there are four different pathways to become a board certified cardiothoracic surgeon in United States (Table 1).

**Table 1:** Training pathways leading to board certification in cardiothoracic surgery in United States

Pathway	Total length of training*	Components	Duration of each component	Board certification
**Classical**	7-8 years	General surgery residency	5 years	General surgery (optional)
	
		Thoracic surgery fellowship	2-3 years	Thoracic surgery

**Fast-track (4+3)**	7 years	General surgery residency	4 years	General surgery (optional)
	
		Thoracic surgery fellowship	3 years	Thoracic surgery

**Integrated**	6 years	Integrated cardiothoracic surgery residency	6 years	Thoracic surgery

**Vascular + Thoracic**	7-8 years	Integrated vascular surgery residency	5 years	Vascular surgery
	
		Thoracic surgery fellowship	2-3 years	Thoracic surgery

* not considering time off for dedicated research or other academic enrichment

• Most common pathway requires successful completion of five-year long general surgery residency, followed by additional two to three years of cardiothoracic surgery fellowship. Board certification in general surgery is not required [1].

• 4/3 joint training pathway requires 4 years of general surgery residency training followed by 2 years of cardiothoracic surgery fellowship, both part of the training has to be completed at the same institution. Board certification in general surgery is allowed after completing 4½ years of general surgery residency, but is not required. Despite the name, total duration of the training is not shortened, it only provides somewhat increased exposure to cardiothoracic surgery compared to the most common pathway.

• Integrated pathway includes six years of dedicated training in cardiothoracic surgery, as well as related surgical and non-surgical specialties. It does include 24 months of core general surgery training, however board certification in general surgery is not allowed.

• Yet another pathway to become a cardiothoracic surgeon is to complete integrated vascular surgery residency (5 years) followed by regular 2-3 year cardiothoracic surgery fellowship [1]. Board certification in vascular surgery is required to enter cardiothoracic surgery fellowship.

Surgical training programs in United States have strictly determined number of categorical positions which ensures that each trainee accepted on a position has enough exposure to all the aspects of the training, including operative experience. Additional work is being taken over by non-categorical trainees and Physician Assistants.

German training in cardiothoracic surgery requires two years of general surgical training ("common trunk") followed by specialty training for additional four years of dedicated training in cardiothoracic surgery [2]. Compared to U.S. training pathways, it is most similar to integrated cardiothoracic surgery residency, however, it has a much stronger component of vascular surgery training. Training in Germany does not have a strict timeframe. It is rather flexible in time and allows to remain in the program for longer time if operative or other requirements are not met. German healthcare system does not have Physician Assistants. As a result, significantly more residents are required on lower level of training than on upper level, and only part of them graduates successfully.

### Certification

In United States, board certification exam in cardiothoracic surgery is administered in two parts: computer-based multiple-choice test questions and oral exam. Board certified cardiothoracic surgeon in United States is eligible to practice both cardiac as well as general thoracic, but not vascular surgery. For vascular surgery, separate board certification is required. In Germany, after all requirements are met, an oral examination is required for board certification. A board certified cardiothoracic surgeon in Germany can practice not only cardiac and general thoracic, but also vascular surgery.

### Operative experience

American Board of Thoracic Surgery requires an average of 125 major operations in each year as a primary surgeon, with a minimal number of 100 in any one year. Based on the length of program, this makes 250 major cases for two-year fellowships and 375 major cases for three-year fellowships. For 4/3 joint training programs the requirement is 250 major cases. For six-year integrated programs, the requirement is 375 major cases (for the last three years of training).

Residents who started training after 07/01/2007 must meet operative requirements for one of two pathways: cardiac or general thoracic surgery. CTSNet is the primary data collection system for case logging. Distribution of cases is outlined in Table 2 for both cardiac as well as general thoracic pathways (255 cases total, corresponding to two-year fellowship).

**Table 2:** Required types and number of cases for cardiac and general thoracic surgery pathways for board certification in United States

Cardiothoracic Pathway	Requirements	General Thoracic Pathway
**20**	**Congenital Heart Disease**	10*
10	Primary	
10	First Assistant	*All cases can be as First Assistant

**150**	**Adult Cardiac**	**75**
50	Acquired Valvular Heart	20
80	Myocardial Revascularization	40
15	Re-Operations	5
5	Aorta	0
15	Other	15

**50**	**Lung, Pluera, Chest Wall**	**100**
30	Pneumonectomy, lobectomy, Segmentectomy	50
20	Other	50

**5**	**Mediastinum (resection)**	**10**

**15**	**Esophagus**	**30**
10	Esophagectomy/Resection	20
0	Benign Esophageal Disease	5
0	Other	5
5	Benign Esophageal Disease/Other	0

**15**	**VATS**	**30**

255	Total	255

40	Endoscopy	90
20	Bronchoscopy	40
10	Esophagoscopy	25
10	Mediastinoscopy	25

100	Consultative Experience	100
50	New Patients	50
50	Follow-up	50

In Germany, number and type of cases are defined by state medical boards. There is, however, no specific number or types of cases defined for each year, which allows training period to be prolonged if needed. Each trainee has a Logbook of Cardiac Surgery which serves as a comprehensive protocol and allows documenting the level of training as well as defines minimum number of operations required for board certification. Required types and numbers of cases for board certification are outlined in Table 3.

**Table 3:** Required types and number of cases for board certification in Germany

Required procedure	Required number of cases
CABG	150

Mitral valve, including reconstruction	10

Aortic valve and ascending aorta/mitral valve/coronary artery	25

Anastomosis and reconstruction of the thoracic vessels, including aortic aneurysms (off bypass)	50

AICD implantation	25

Thoracic operations related to cardiac surgery procedures, e.g. chest wall resection, thorax stabilisation, extripation of foreign bodies, operations for thoracic injuries	10

Pulmonary operations and the bordering mediastinum in relation to cardiac surgery operations	10

Operations on peripheral vessels in relation to cardiac surgery procedures, e.g. reconstruction of peripheral vessels after application of circulatory assist systems/extracorporal circulation	50

Application and supervision of extracorporal circulation and circulatory assist systems	50

Application of diagnostic procedures, intubation, application of central venous catheters, arterial cannulation, application of thoracic drains, puncture of pleura, pericardium and lungs	150

**Figure 1:**
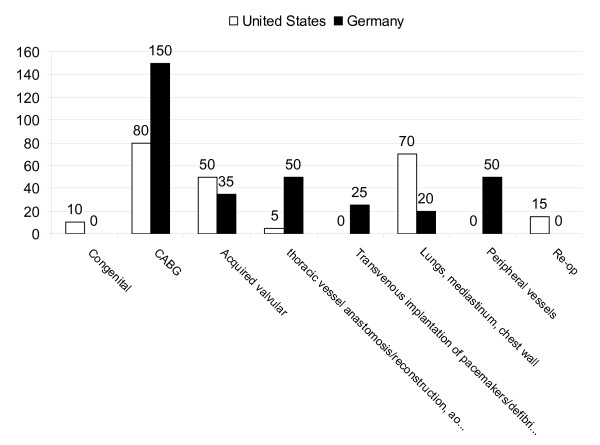
**Quantitative comparison of case requirements by U.S. and German medical boards**. To create similar categories, certain case groups have been merged into larger groups.

Quantitative comparison of case requirements by U.S. and German boards (Figure 1) shows that the American Board of Thoracic Surgery requires more general thoracic cases than German State Medical Boards do. On the other hand, German State Medical Boards require more coronary artery bypass grafting and peripheral vascular cases than American Board of Thoracic Surgery does.

### Non-operative clinical requirements

Non-operative clinical requirements are similar in USA and Germany and include pre- and post-operative care, ICU and ward experience, as well as consultations. Physician Assistant as a profession does not exist in Germany which is counterbalanced by higher number of junior residents than senior residenets. This could make it more challenging to balance operative and non-operative experience.

### Non-clinical academic enrichment

To perform non-clinical academic work, e.g. high-quality research, time is of great importance in recent days especially for young residents [3]. Therefore, many trainees in U.S. hold their training after 2^nd ^or 3^rd ^year of general surgery residency and perform one to three years of dedicated research during General Surgery residency. According to a recent national survey, 36% of general surgery residents interrupt residency to pursue full-time research, with mean research fellowship length of 1.7 years, and with 72% of research fellows performing basic science research [4-6].

In Germany there is no dedicated research time taken off during the training. Most trainees at university hospitals perform successful research simultaneously with their clinical training which is easier in Germany given more flexible duration of training.

### Salary

The salary in USA is based mainly on post-graduate year and does not depend on the specialty a person is being trained in. Below is a table with nationwide resident/fellow salaries for the 2008-2009 academic year (Table 4) [4]. The annual salary for a U.S. cardiothoracic surgeon ranges from $245.000 to $621.000 [5].

**Table 4:** Annual resident/fellow salaries for the 2008-2009 academic year, published by the Association of American Medical Colleges (AAMC) 5.

Post-MD Year	N	Mean	25^th ^Percentile	50^th ^Percentile	75^th ^Percentile
1	210	$46,245	$44,055	$45,659	$47,760

2	213	48,092	45,720	47,257	49,764

3	213	50,128	47,290	49,095	51,857

4	212	52,154	48,911	50,987	54,468

5	199	54,164	50,606	52,956	56,451

6	182	56,463	52,746	55,265	59,282

7	152	58,520	54,147	57,027	62,520

8	85	60,278	55,266	59,108	63,825

The salary structure of German cardiac surgery trainees is also based on the number of post-graduate years completed (Table 5). The salary itself is the same for German surgery residents nationwide.

**Table 5:** Monthly salary of residents in Germany

Post-Graduate Year (not board certified)	Amount in EURO's
1	EUR 3,705

2	3,915

3	4,065

4	4,325

5	4,635

Years after board certification	

1-3	4,890

4-6	5,300

7 and above	5,660

Years after becoming an attending surgeon	

1-3	6,125

4-6	6,485

7 and above	7,000

Comparison in financial compensation between USA and Germany would be biased and is not performed intentionally. The bias is multifactorial and most importamtly includes different cost of living, costs of insurancies, different education system (public vs private), and also different currencies in USA and Germany. However, it can be noted that change from a trainee status to an attending status is followed by a bigger jump in financial compensation in USA than in Germany.

### Job satisfaction

Overall dissatisfaction among cardiothoracic surgery graduates is similar in USA and Germany. This is most likely attributed to the minimal number of available jobs open, low reimbursements and lifestyle issues [7,8]. Annual reports of National Resident Matching Program show that the number of applicants in United States interested in cardiothoracic surgery training are steadily declining (Table 6) [9]. In Germany, overall situation is very similar. A special committee of German Society for Cardiac, Thoracic and Vascular Surgery (GSCTS) conducted an inquiry of young trainees wich revealed the following:

**Table 6:** National Resident Matching Program thoracic surgery match data from 1996 to 2008 8.

	1996	1997	1998	1999	2000	2001	2002	2003	2004	2005	2006	2007	2008
Certified positions	146	143	138	137	139	141	144	144	141	138	139	126	130

Certified applicant	197	176	175	156	156	148	149	145	161	134	104	91	96

Programs filled (%)	93.5	88.0	94.7	91.1	89.1	94.5	88.4	84.0	92.6	81.7	67.4	63.0	60.9

Positions filled (%)	95.9	92.3	96.4	93.4	92.1	95.7	91.0	85.4	93.6	87.7	71.9	66.7	66.9

Matched applicants (%)	71.1	75.0	76.0	82.1	82.1	91.2	87.9	84.8	82.0	90.3	96.2	92.3	90.6

Unmatched applicants (%)	28.9	25.0	24.0	17.9	17.9	8.8	12.1	15.2	18.0	9.7	3.8	7.7	9.4

Certified positions filled with US grads (%)	80.8	76.9	77.5	73.0	69.1	73.8	70.8	65.3	75.9	66.7	49.6	47.6	47.7

• It is currently impossible to staff all positions in cardiac surgical hospitals. An average of 1.2 positions per hospital is available.

• The majority of members are not satisfied with their situations.

• Partial payment for overtime occurs in only 73% of evaluated hospitals.

• Of particular note, almost 70% of residents in cardiac surgery are not satisfied with current compensation.

• Despite the introduction of a new theoretical concept for post-graduate training and creation of a logbook, a well structured concept for post-graduate training exists in only 29% of hospitals.

• The average age at the time of board certification is 36.6 years. Overall, there exists considerable discontent regarding post-graduate training (only 27% of responses are satisfactory).

• Women are a minority in cardiac surgery - only 24% amongst residents.

• In Germany, cardiac surgery has traditionally been an international specialty. One quarter of all colleagues represents foreign medical graduates - most from countries not part of the European Union. 90% of staff members are salaried whereas 10% are financed by scholarships.

## Discussion

Both the United States and German cardiac surgery training programs have their own advantages and disadvantages. It will be useful to consider each other's advantages to attract well-qualified individuals. Building an internationally comparable efficient cardiothoracic surgical program should have the same principles and values as a traditional institutional or single country program: high-quality patient care, training and fostering residents and contributing to basic and clinical research. Lot of questions remain to be answered: For example, is it still necessary to be trained in general surgery before becoming a cardiothoracic surgeon? If so, how many years of general surgery are really necessary prior to starting a cardiothoracic surgery training program? The best decision for now seems to keep open diverse training pathways, leading to thoracic surgery certification, and with time we will determine which way is superior to attract best candidates and train best surgeons in a constantly changing environment.

## Conclusions

1. Both, the United States and German Cardiac Surgery Training Programs have their own advantages and disadvantages.

2. Training in Germany is similar to a pyramidal system and creates a strong competition inside the program. In USA, most of the competition between applicants takes place before entering the program in USA, rather than inside the program.

3. Training in Germany is more flexible and does not have a strict timeframe compared to the training in USA.

4. Lack of Physician Assistant profession in Germany could make it more challenging to balance operative and non-operative experience for a trainee.

5. Research training in USA is mostly performed as dedicated 1-3 years in a research laboratory. In Germany, research training takes place simultaneously with clinical training. This is facilitated by flexibility of training in Germany.

6. Change from a trainee to an attending level is followed by a bigger jump in financial compensation in USA than in Germany.

7. Work hour restrictions in Germany exceed work hours restrictions in USA.

8. Training in Germany has a much stronger component of vascular surgery training compared to the training programs in USA.

9. At this time, there is equal job dissatisfaction among graduates of cardiothoracic surgery training in both USA and Germany.

## Competing interests

The authors declare that they have no competing interests.

## Authors' contributions

VT conceived the study, provided the information on cardiothoracic surgery training in USA, participated in literature search, drafted the manuscript. SM participated in drafting the manuscript. TKR provided the information on cardiothoracic surgery training in Germany, participated in literature search and drafting the manuscript. RMB participated in drafting the manuscript, supervised and reviewed the manuscript. FYC supervised the work, provided information on cardiothoracic surgery training in USA, participated in drafting the manuscript, reviewed the manuscript. JDS provided the information on cardiothoracic surgery training in Germany, participated in drafting the manuscript, participated in literature search, reviewed the manuscript, participated in its design and coordination. All authors read and approved the final manuscript.
